# ADVANCE CARE PLANNING IN PRIMARY CARE: ENGAGING ACCOMPANIED OLDER ADULTS WITH COGNITIVE IMPAIRMENT

**DOI:** 10.1093/geroni/igad104.0039

**Published:** 2023-12-21

**Authors:** Jennifer Wolff, Diane Echavarria, Laura Gitlin

## Abstract

Alzheimer’s Disease and Related Dementias (ADRD) are among the most profoundly disabling and costly of all health conditions, and persons living with dementia are at heightened risk for high utilization of burdensome and costly end-of-life care. Family caregivers are at the forefront of managing ADRD, however, they are not routinely engaged in primary care discussions about prognosis and the healthcare preferences of the people for whom they care. Advance care planning (ACP) is a longitudinal communication process that supports adults at any age or stage of health in understanding and sharing their personal values, life goals, and preferences regarding future medical care. Early initiation of ACP, communication with the primary care team, and the inclusion of associated caregivers is imperative in ADRD care due to progressive and devastating effects on decision-making capacity connected with the disease. SHARE, a multicomponent communication intervention, engaged 273 patient-family caregiver dyads in a randomized-controlled trial collecting baseline and six-month survey data related to experiences in the primary care setting. 145 patient-caregiver dyads assigned to the intervention received an offer for a facilitated advance care planning conversation. This symposium will highlight 6-month outcomes from caregiver-reported surveys. Attendees will gain a perspective on ACP considerations in a cognitively impaired population including ethical considerations and the factors influencing preparedness for medical decision making for involved family caregivers. Additional attention concerning the quality and content of ACP conversations with patient-caregiver dyads and the development of a fidelity tool for usage with audio-recorded ACP conversations will be presented.

